# Spatial encoding and growth-related change of sheep lung radiomic features

**DOI:** 10.3389/fvets.2025.1495278

**Published:** 2025-02-26

**Authors:** David Collie, Chris Cousens, Steven Wright, Ziyuan Chang, James Meehan, Helen Brown, Calum D. Gray, Tom J. MacGillivray, David J. Griffiths, Chad E. Eckert, Nicole Storer, Mark Gray

**Affiliations:** ^1^The Roslin Institute, Royal (Dick) School of Veterinary Studies, University of Edinburgh, Edinburgh, United Kingdom; ^2^Moredun Research Institute, Pentlands Science Park, Penicuik, United Kingdom; ^3^Edinburgh Imaging Facility, Queen's Medical Research Institute, University of Edinburgh, Edinburgh, United Kingdom; ^4^Centre for Clinical Brain Sciences, College of Medicine and Veterinary Medicine, University of Edinburgh, Edinburgh, United Kingdom; ^5^Interventional Oncology, Johnson & Johnson Enterprise Innovation, Inc., New Brunswick, NJ, United States

**Keywords:** lung, computed tomography, radiomic features, lobar variation, sheep

## Abstract

**Introduction:**

Different regions of the small ruminant lung exhibit variable susceptibility to specific lung pathologies. Such susceptibility may be reflected in regional lung radiomic features extracted from computed tomography (CT) images. In this study, we investigated whether region-specific variation in radiomic features exists in ovine lungs and whether these features remain stable over time.

**Methods:**

Thoracic CT image datasets from 30 young adult sheep were subject to an image segmentation protocol directed at partitioning the lung into individual lobar and sub-lobar segments for radiomic feature analysis. After identifying and removing unstable, non-reproducible, and highly correlated features, 22 features remained and were used as input for principal component (PC) analysis.

**Results:**

The significance of segment-related influence on PC scores was determined and visualised. For six sheep, successive CT images were acquired at monthly intervals for a period of 9 months in order to assess time-dependent variation in radiomic features. The results indicated that there was a significant difference in radiomic features derived from different lung segments. Visualisation of PC scores highlighted differences between caudodorsal and cranioventral lung, between lobar and sub-lobar segments, and suggested a bias towards one lung or the other. Significant changes in PC scores occurred over time. With few exceptions, largely similar changes occurred across all segments in this regard.

**Discussion:**

Overall, our results indicate that although sheep lung radiomic features are influenced by the lung segment of origin, their variation over time is largely consistent throughout the lung. Such influence should be borne in mind when interpreting radiomic features and their changes over time.

## 1 Introduction

Respiratory disease in cattle and sheep is usually caused by a variety of pathogens, both viral (bovine respiratory syncytial virus (BRSV), parainfluenza 3 (PI3), adenovirus, bovine viral diarrhoea virus (BVDV), and infectious bovine rhinotracheitis (IBR)) and bacterial (*Pasteurella multocida, Mannheimia haemolytica, Histophilus somni, and Mycoplasma bovis*). Bovine respiratory disease is estimated to cost the UK £60–80 million annually (1, 2) and the EU €576 million (3), with losses related to mortality, treatment costs, and reduced performance both during and after the period of illness. In North America, bovine respiratory disease is the most prevalent and economically devastating health concern of the cattle industry with the overall cost to the industry estimated, in 1997, at over $750 million per year (4). Global losses are estimated to be >$3 billion/year (5).

Ruminants display a characteristic regional susceptibility to lung pathology. Indeed, the disease associated with *Pasteurella multocida* and *Mannheimia haemolytica* infection typically displays a cranioventral distribution with often a sharp line of demarcation between the affected tissue and grossly normal tissue located caudodorsally. Whilst gravitational influence on inhaled bioaerosols has been speculated to contribute to this distribution (6), a similar distribution of cranioventral lung lesions is seen following intravenous inoculation of calves with *Pasteurella haemolytica* (7). Furthermore, intravenous endotoxin administration in supine sheep results in atelectasis of dependent lung regions and an associated increase in cellular pulmonary metabolism (8, 9). This effect is assumed to be reflective of an influx and activation of inflammatory and immune cells. Therefore, in small ruminants, the cranioventral lung regions appear particularly susceptible to infection, whether from blood or airborne sources. Understanding the basis of such regional susceptibility would be a key factor in preventing or mitigating the impact of respiratory disease.

In contrast to the radiologic interpretation of lung disease, which is primarily based on a qualitative assessment of first-order features reflecting the extent of attenuation and recognising the morphological patterns that are often associated with signs, symbols, or naturalistic images (10), radiomic feature analysis extracts measures of the spatial relationships between pixel intensities. In particular, radiomic feature analysis offers the facility to quantify aspects of largely ‘subvisual’ image texture. Such measurements can potentially provide insight into the histology and biology of the associated tissue (11, 12). Radiomics is a rapidly evolving field, particularly in the area of characterisation and monitoring of lung cancer (13, 14), where measurements are of notable value in the context of predicting the malignant and metastatic potential of lung tumour nodules and the response to treatment (15–17).

Whilst associations between global measures of lung function and ‘lung-wide’ radiomic feature characteristics have been described (18), it is only more recently that the potential of radiomic feature analysis to quantify subregional pulmonary function has been explored (19). The latter study found that certain radiomic features (GLRLM run length non-uniformity and GLCM sum average) were highly correlated with functional imaging of regional ventilation. Subregions within the lung could also be described as habitats, acknowledging that variations in form and function do exist throughout the whole organ.

With the long-term goal of better understanding the factors underlying regional susceptibility to lung disease processes in small ruminants, we initially sought to address the primary hypothesis that radiomic features are spatially encoded in the healthy ovine lung. To this end, we developed an image characterisation workflow protocol to facilitate radiomic feature extraction. We used this to demonstrate region-specific variation in radiomic features and further demonstrated the stability of such features over time in growing sheep.

## 2 Materials and methods

### 2.1 Animals

Studies were performed under a UK Home Office Project Licence in agreement with the Animals (Scientific Procedures) Act 1986 and with consent from the University of Edinburgh Animal Welfare and Ethical Review Body. The recommendations for the welfare and use of animals in research were adhered to.

Details of the animals used, together with the overarching experimental design, are elaborated in a previous publication (20). Briefly, in addition to baseline computer tomography (CT) data obtained from 30 animals in order to verify lung health, data from CT scans repeated on a monthly basis over a period of 9 months were also obtained from six control animals from that cohort. Thirty young adult sheep (~4mo Texel cross mule; 39.2 ± 4.8 kg (mean ± SD) of mixed sex (17 female and 13 male neutered) were commercially sourced. Sheep were housed on straw bedding under standard management conditions appropriate to a research setting. All imaging procedures were conducted under general anaesthesia, managed by veterinary specialist anaesthetists. Pre-anaesthetic medication, analgesics, induction, and maintenance of anaesthesia were performed as previously reported (20).

### 2.2 CT acquisition

A multislice SOMATOM Definition AS 64 slice helical CT machine was used to obtain thoracic CT scans (Siemens Healthcare Ltd., Erlangen, Germany) from each prone-positioned sheep. For six sheep, a further eight thoracic CT scans were acquired at monthly intervals to assess the nature and extent of time-dependent variation in radiomic features. An incremental continuous positive airway pressure (CPAP) protocol was applied to induce apnoea and standardise lung volume for CT.

### 2.3 Image processing

DICOM images were imported for segmentation in 3D Slicer software version 4.11.2.^1^ Lungs were segmented by visual assessment using typical window threshold levels between −1,024 and − 240 HU, with subsequent island selection and trimming as appropriate. Following airway segmentation (typical threshold levels between −1,024 and − 900 HU), new segmentations were created and named according to the bronchial anatomy as described by Hare (21). The 3D paint facility was used to paint spheres centred on the relevant bronchi, and the ‘grow from seeds’ effect within the segment editor used the fast grow-cut method to simultaneously grow the spheres to neighbour boundaries within the lung segmentation. The radiomics module was applied to measure the radiomic features of the lung segments, selecting all features (resampled voxel size = 2,2,2, bin width = 64, enforced symmetrical GLCM) and saving the results to a file. Features used in subsequent analysis are indicated in Table 1. Radiomic feature maps were prepared using the graphical user interface developed by Kim et al. (22).

**Table 1:** Radiomic features extracted using the radiomics module in Slicer.

Feature Class	Features
First order	10th percentile, 90th percentile, energy, entropy, interquartile range, kurtosis, maximum, mean absolute deviation, mean, median, minimum, range, robust mean absolute deviation, root mean squared, skewness, total energy, uniformity, variance
GLCM—Grey-level co-occurrence matrix	Autocorrelation, cluster prominence, cluster shade, cluster tendency, contrast, correlation, difference average, difference entropy, difference variance, inverse difference (Id), inverse difference moment (idm), inverse difference moment normalised (IDMN), inverse difference normalised (Idn), informational measure of correlation (IMC) 1, informational measure of correlation (IMC) 2, inverse variance, joint average, joint energy, joint entropy, maximal correlation coefficient (MCC), maximum probability, sum average, sum entropy, sum of squares
GLDM—Grey-level dependence matrix	Dependence entropy, dependence non-uniformity, dependence non-uniformity normalised, dependence variance, grey-level non-uniformity, grey-level variance, high grey-level emphasis, large dependence emphasis, large dependence high grey-level emphasis, large dependence low grey-level emphasis, low grey-level emphasis, small dependence emphasis, small dependence high grey-level emphasis, small dependence low grey-level emphasis
GLRLM—Grey-level run length matrix	Grey-level non-uniformity, grey-level non-uniformity normalised, grey-level variance, high grey-level run emphasis, long run emphasis, long run high grey-level emphasis, long run low grey-level emphasis, low grey-level run emphasis, run entropy, run length non-uniformity, run length non-uniformity normalised, run percentage, run variance, short-run emphasis, short-run high grey-level emphasis, short-run low grey-level emphasis
GLSZM—Grey-level size zone matrix	Grey-level non-uniformity, grey-level non-uniformity normalised, grey-level variance, high grey-level zone emphasis, large area emphasis, large area high grey-level emphasis, large area low grey-level emphasis, low grey-level zone emphasis, size zone non-uniformity, size zone non-uniformity normalised, small area emphasis, small area high grey-level emphasis, small area low grey-level emphasis, zone entropy, zone percentage, zone variance
NGTDM—Neighbouring grey tone difference matrix	Busyness, coarseness, complexity, contrast, strength

First-order statistics describe the distribution of voxel intensities, grey-level co-occurrence matrix (GLCM) features quantify image texture by calculating how often pairs of pixels with specific values and in a specified spatial relationship to each other occur, grey-level dependence matrix (GLDM) features quantify grey-level dependencies defined by the number of connected voxels within a given distance that are dependent on the centre voxel, grey-level run length matrix (GLRLM) features quantify the occurrence of grey-level runs of consecutive pixels having the same value in a particular direction, grey-level size zone matrix (GLSZM) features quantify grey-level zones defined as the number of connected voxels that share the same grey-level intensity, and neighbouring grey tone difference matrix (NGTDM) features quantify the difference between a grey value and the average grey value of its neighbours within a certain distance.

### 2.4 Assessment of reproducibility and stability

Reproducibility, defined by Tunali et al. (23) as the “consistency of a feature across image acquisition parameters such as patient position and respiration phase,” was assessed using paired thoracic CT image datasets acquired from one sheep before and after minor repositioning. Stability, defined as the “consistency of a feature across different segmentations,” was assessed by comparing feature measurements derived from separate segmentations applied to the same DICOM image series, from one sheep, and implemented by the same operator (DC) on two separate occasions. Features failing to demonstrate significant association between datasets were excluded from further analysis.

### 2.5 Removal of redundant features

In order to facilitate steps to reduce the dimensionality of the dataset, blocks of highly correlated variables (correlation coefficient > 0.95) were identified and redundant features were removed from each block to leave only one feature with the highest variance. The R corrplot package (24) was used for the correlation analysis.

### 2.6 Statistical analysis

Statistical analysis was conducted using Minitab (Minitab 20, www.minitab.com). Principal component analysis (PCA) was applied as an initial exploratory tool to determine the features contributing the most to respective principal components (PCs) and to assess whether anatomical disposition had a bearing on PC scores. PCA was applied to the z-score normalised radiomic features derived from each initial CT scan, and the PCs explaining the majority of the variance retained. ANOVA (mixed-effects model with sheep as a random factor and segment as a fixed factor) was applied to the first PC to determine whether the segment explained a significant proportion of variance (25). Manual colour selection of Microsoft Excel conditional formatted PC score values and application to lung segmentations in Slicer facilitated the visualisation of PC score lung segmental distribution. In addition, segmentations representing caudodorsal to cranioventral lung slices were prepared and used to mask and crop lung volumes. These cropped volumes, exported in DICOM format, were used as input into the graphical user interface developed by Kim et al. (22), with a voxel region of interest of 3 × 3 × 3, and radiomic feature classes selected as appropriate. In order to assess the time-dependent variation in PC scores for six sheep over the course of 9 months, the eigenvectors derived above were applied to the relevant z-score normalised radiomic features from the repeated data. One-way ANOVA was used to determine whether a significant change occurred over time for individual lung segments, and the correlation between PC scores derived from different lung segments was visualised through correlograms.

## 3 Results

### 3.1 Lung segmentation protocol

**Figure 1:**
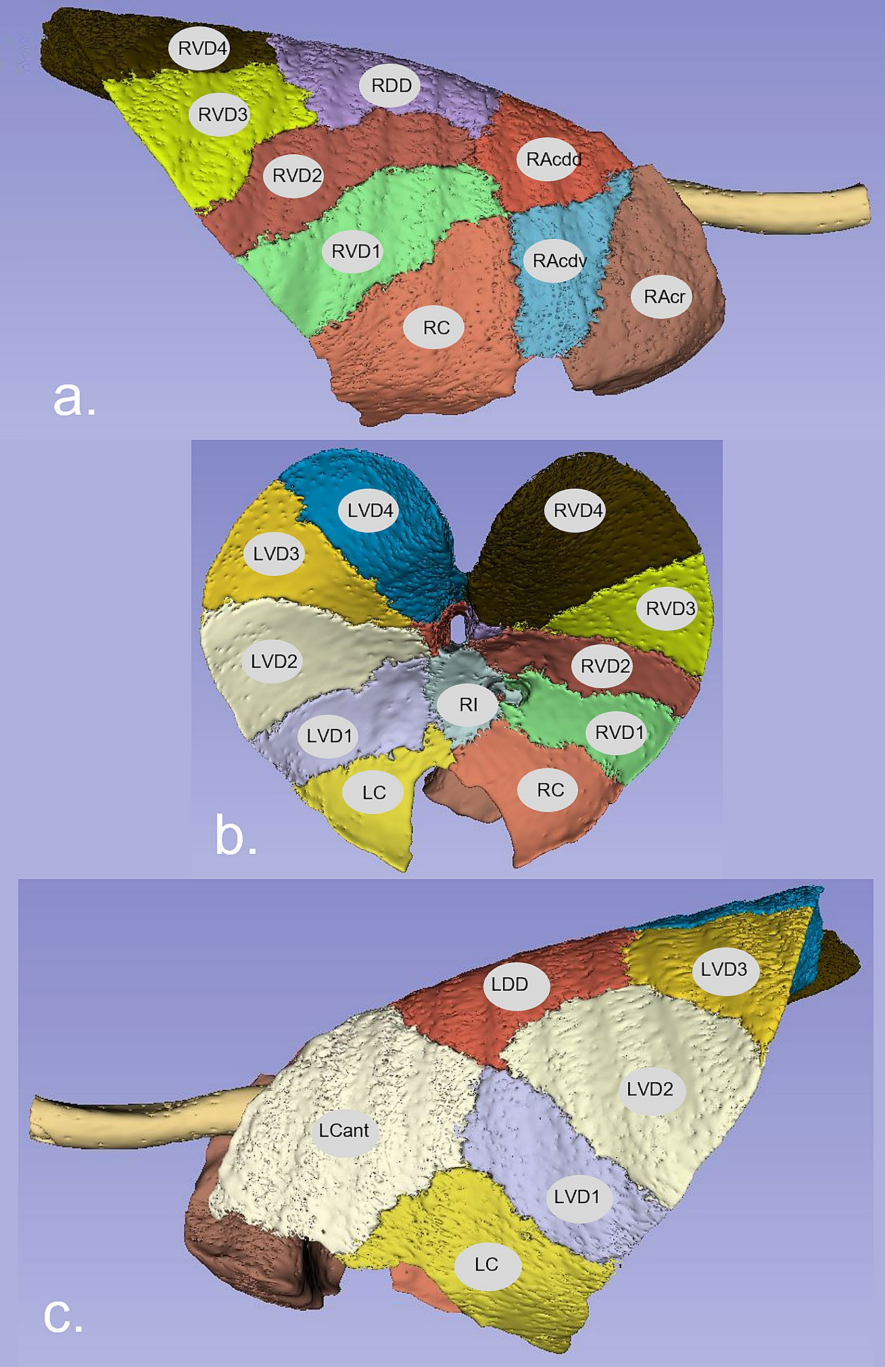
Representation of the pleural surface of sheep lung segments. A manual segmentation protocol based on the disposition of segmental and sub-segmental bronchi was applied. The lung segment nomenclature is based on the bronchial anatomy as described by Hare (21). **(A)** Right lateral view, **(B)** ventral view, and **(C)** left lateral view. RAcr—Cranial segmental bronchus of the right apical lobe; RAcdv—ventral branch of caudal segmental bronchus of the right apical lobe; RAcdd—dorsal branch of caudal segmental bronchus of the right apical lobe; RC—right cardiac lobar bronchus; RI—right intermediate lobar bronchus; RVD1/2/3/4—1st, 2nd, 3rd, and 4th ventral basal segmental bronchus of the right diaphragmatic lobe; RDD—right dorsal diaphragmatic; LCant—apical segmental bronchus of the left apico-cardiac lobe; LC—left cardiac lobar bronchus; LVD1/2/3/4—1st, 2nd, 3rd, and 4th ventral basal segmental bronchus of the left diaphragmatic lobe; LDD—left dorsal diaphragmatic.

Visual appraisal of the results of the lung segmentation protocol is shown in Figure 1, where the pleural margins of the various lung segments can be discerned. The various segmental dispositions are largely consistent with the authors’ experience in relation to segmental bronchoalveolar lavage procedures applied to ovine lungs post-mortem, where the expansion of those individual segments can be visualised (DC, personal communication).

### 3.2 Reproducibility and stability

The radiomic features as listed in Table 1 proved consistent across different segmentations, with a median correlation of 0.94, and all features demonstrate a significant correlation (*p* < 0.05). Reproducibility of the features on two separate scans following minor repositioning proved less consistent (r^2^ 0.88) and 12 features failed to demonstrate a statistical association between repeat measurements on CT retake. Following their removal and the subsequent removal of features within the dataset that were highly correlated, a total of 22 features remained.

### 3.3 Principal component analysis

**Figure 2:**
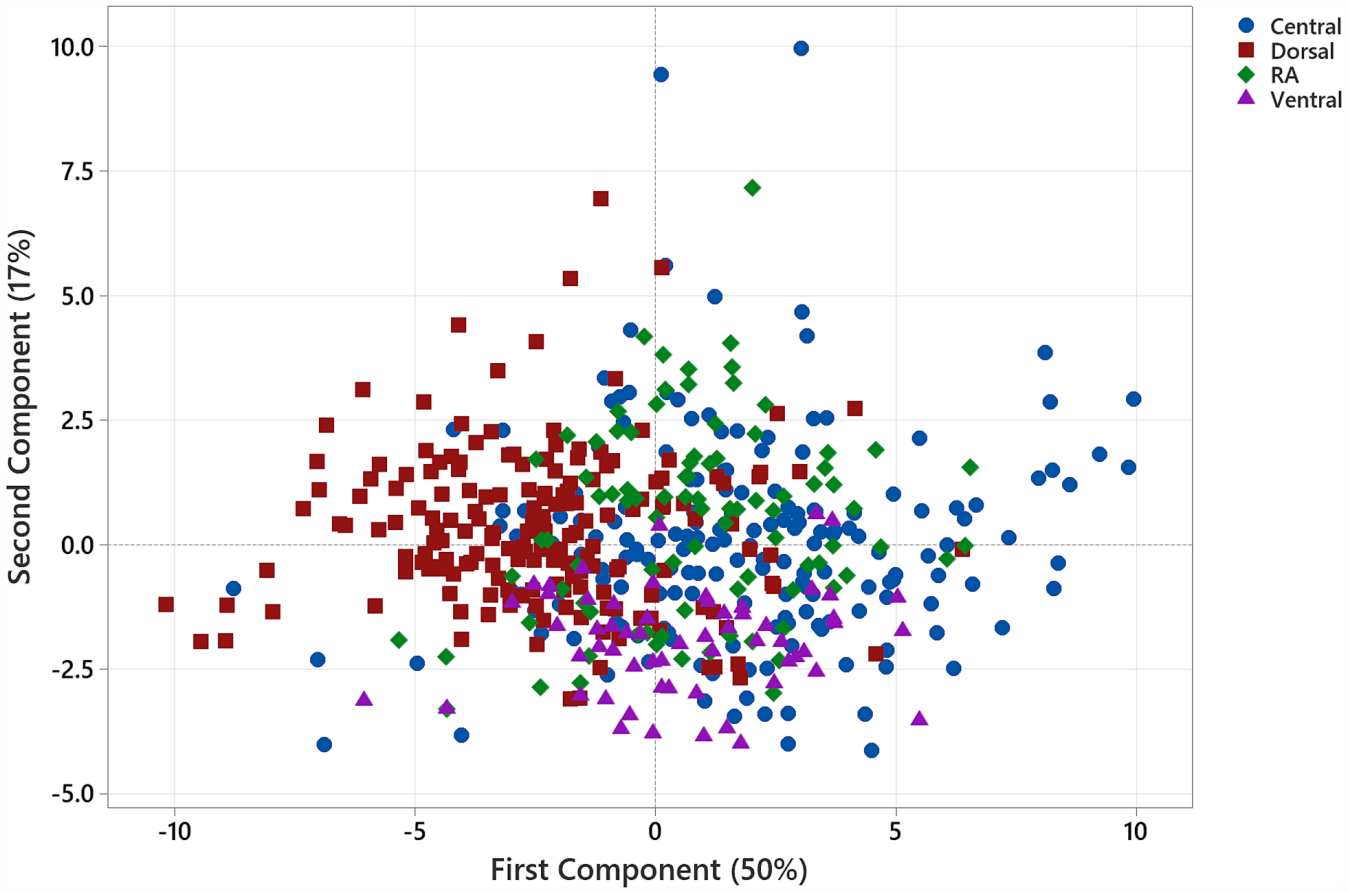
Score plot for the first and second principal components illustrating clustering according to the spatial disposition of lung segments within the chest. Markers are coloured according to segment position within the chest (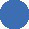 Central: LCant, LVD1, LVD2, RI, RVD1, and RVD2; 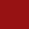 Dorsal: LDD, LVD3, LVD4, RDD, RVD3, and RVD4; 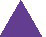 Ventral: LC & RC; 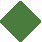 RA). Dorsal segments are clustered to the left of the origin of the first component axis.

PCA applied to the 22 features using a correlation matrix indicated that 90% of the variance was explained by the first five components (PC1: 50%, PC2: 17%, PC3: 11%, PC4: 8%, and PC5: 4%). Examination of the PC1 vs. PC2 score plot suggested clustering on the first component axis according to whether segments were positioned dorsally (LDD, LVD3, LVD4, RDD, RVD3, and RVD4) or centrally (LCant, LVD1, LVD2, RI, RVD1, and RVD2) in the chest (Figure 2; abbreviations are as listed in the legend).

**Figure 3:**
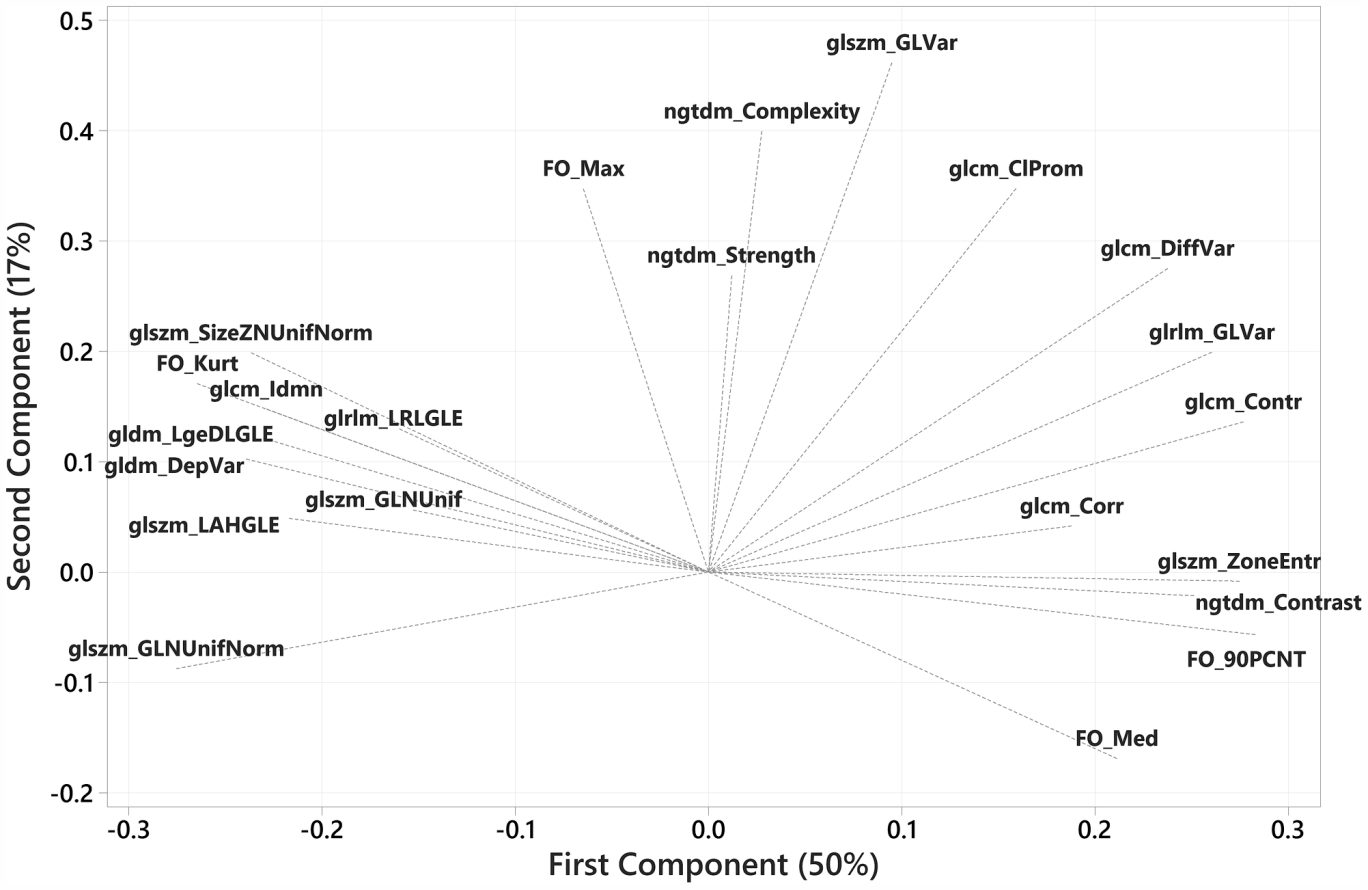
PCA loading plot reflecting feature contributions to the first and second principal components. The first-order features—90th percentile and median, and GLCM—contrast, correlation, and difference variance, GLSZM—zone entropy, and GLRLM—grey-level variance—share strong positive associations, whereas the first-order feature—kurtosis, and the texture features GLCM—Idnm, GLSZM—size zone non-uniformity normalised, large-area high grey-level emphasis and grey-level non-uniformity normalised, and GLDM—large-dependence low grey-level emphasis and dependence variance share strong negative associations with the first component. The majority of features share a positive association with the second principal component, with the first-order feature—median, the most negatively associated with PC2.

The loading plot for the first- and second-order components indicated that the first-order features—90^th^ percentile and median, GLCM—contrast, correlation, and difference variance, GLSZM—zone entropy, and GLRLM—grey-level variance—shared strong positive associations. In contrast, the first-order feature—kurtosis, and the texture features GLCM—Idnm, GLSZM—size zone non-uniformity normalised, large-area high grey-level emphasis and grey-level non-uniformity normalised, and GLDM—large-dependence low grey-level emphasis, and dependence variance—shared strong negative associations with the first component (Figure 3). The eigenvectors for the PCs are provided in Table 1.

### 3.4 Spatial encoding of radiomic features

The results of ANOVA applied to the first PC determined that at least one segment was different from the others (*p* < 0.0001), and the r^2^(adj) value indicated that the ANOVA model explained 77% of the variation in PC1 (Table 2).

**Table 2:** Eigenvectors from PCA.

Feature class	Radiomic feature	PC1	PC2	PC3	PC4	PC5
First Order	90th percentile	0.283	−0.057	−0.115	0.014	−0.152
GLCM	Contrast	0.277	0.136	0.025	−0.033	0.184
GLSZM	Zone entropy	0.275	−0.008	−0.122	0.151	0.065
GLRLM	Grey-level variance	0.261	0.199	0.159	0.086	−0.019
NGTDM	Contrast	0.251	−0.022	0.276	−0.003	0.068
GLCM	Difference variance	0.238	0.275	0.096	−0.021	0.07
First Order	Median	0.211	−0.169	−0.268	−0.101	−0.213
GLCM	Correlation	0.188	0.042	0.232	0.308	−0.5
GLCM	Cluster prominence	0.159	0.348	0.27	0.124	−0.122
GLSZM	Grey-level variance	0.095	0.462	0.027	0.053	0.048
NGTDM	Complexity	0.028	0.399	−0.345	0.045	0.19
NGTDM	Strength	0.012	0.269	−0.065	−0.544	−0.244
First Order	Maximum	−0.065	0.347	−0.393	0.011	−0.037
GLSZM	Grey-level non-uniformity	−0.153	0.056	−0.032	0.562	0.346
GLRLM	Low grey-level run emphasis	−0.16	0.13	0.378	−0.191	0.365
GLSZM	Large-area high grey-level emphasis	−0.217	0.049	−0.071	0.397	−0.157
GLCM	Inverse difference moment normalised	−0.229	0.148	−0.304	0.047	−0.182
GLDM	Large-dependence low grey-level emphasis	−0.23	0.121	0.312	−0.059	−0.054
GLSZM	Size zone non-uniformity normalised	−0.237	0.199	0.096	−0.027	−0.126
GLDM	Dependence variance	−0.239	0.102	0.177	0.112	−0.433
First Order	Kurtosis	−0.265	0.171	−0.007	−0.103	0.034
GLSZM	Grey-level non-uniformity normalised	−0.275	−0.088	0.061	−0.087	0.025

The eigenvectors are linear combinations of the original variables that essentially describe how each variable contributes to each principal component.

**Figure 4:**
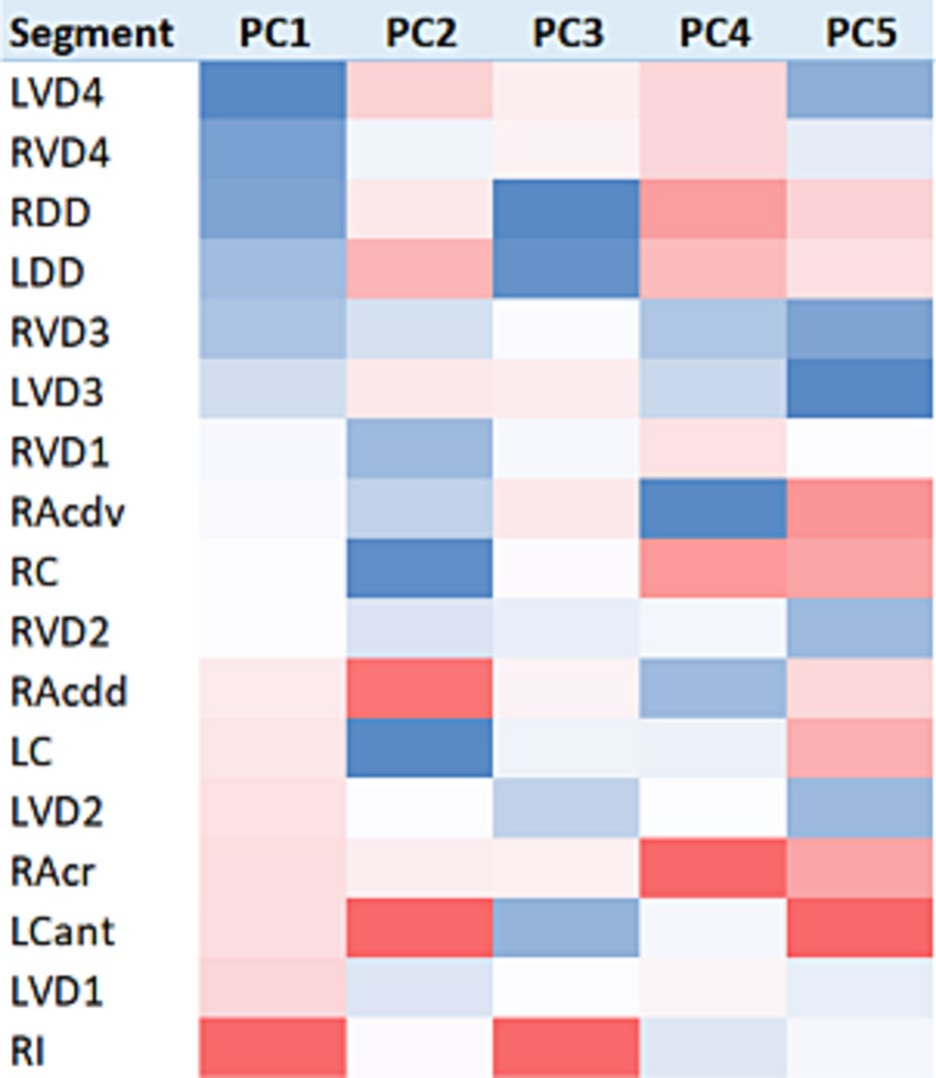
Heatmap visualisation of lung segment PC score data. Baseline (n = 30) PC scores were feature-standardised and conditionally formatted within columns to illustrate the intensity and range of values for each PC score. All columns are sorted on the basis of the first principal component.

**Figure 5:**
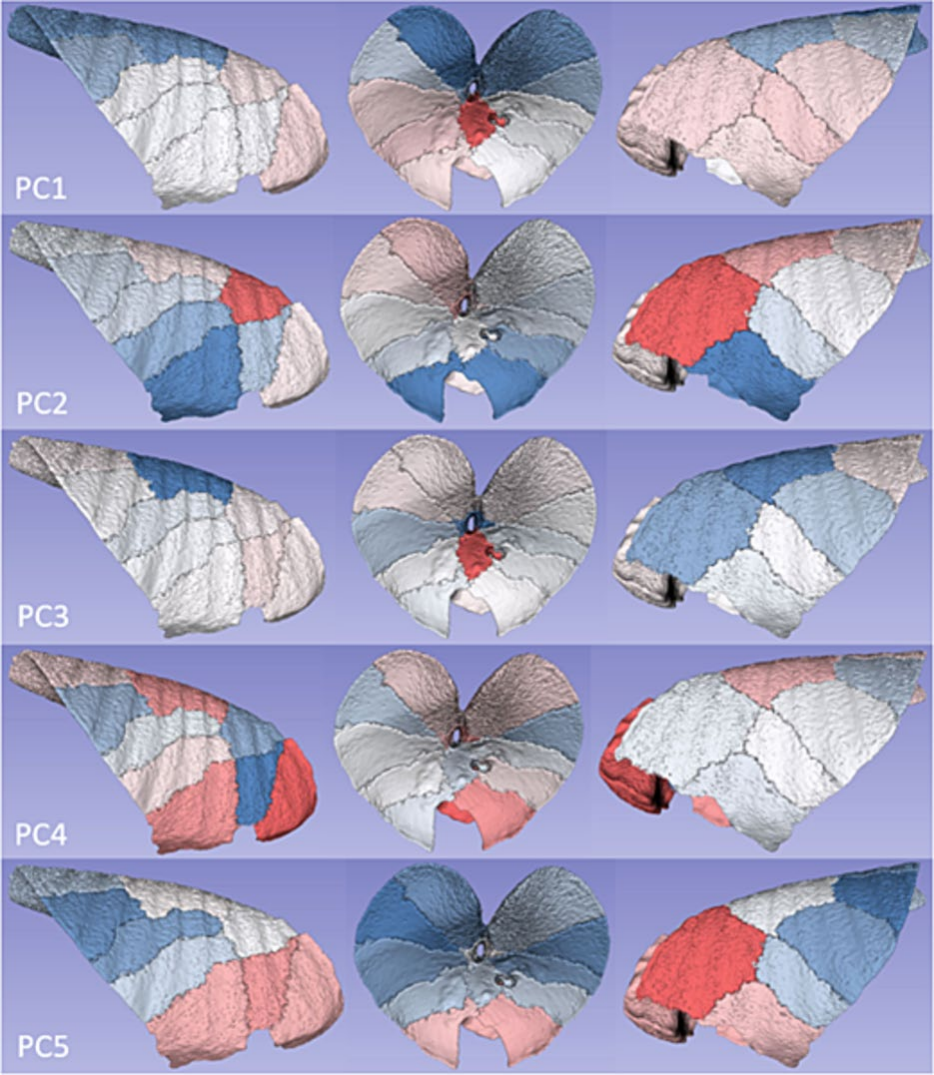
Segmental disposition of PC scores. Colour intensities from the heatmap in Figure 4 were ascribed to the respective segment volumes in order to illustrate the anatomical disposition of average principal component values.

Feature standardisation of the PC score data, sorting based on the scores, and applying conditional formatting to indicate the range and distribution of PC scores served to illustrate the influence of the segment on PC score values (Figure 4). The anatomical disposition of these segmental PC scores is shown in Figure 5.

For the first PC, a gradient of increasingly positive values was apparent going from caudodorsal to cranioventral, with the most positive values present in RI, and some suggestion of a left-sided lung bias towards more positive PC score values. For PC2, cranial and craniodorsal positivity was noted, with markedly negative values in RC and LC. For PC3, strong positivity in RI was apparent, with marked dorsal (LDD and RDD) and craniodorsal (LCant) negativity. PC4 featured strong positivity in RAcr and positivity in RC, RDD, and LDD. For PC5, an increasingly positive value gradient was apparent going from caudodorsal to cranioventral in both lungs, with the most positive values present in LCant.

**Figure 6:**
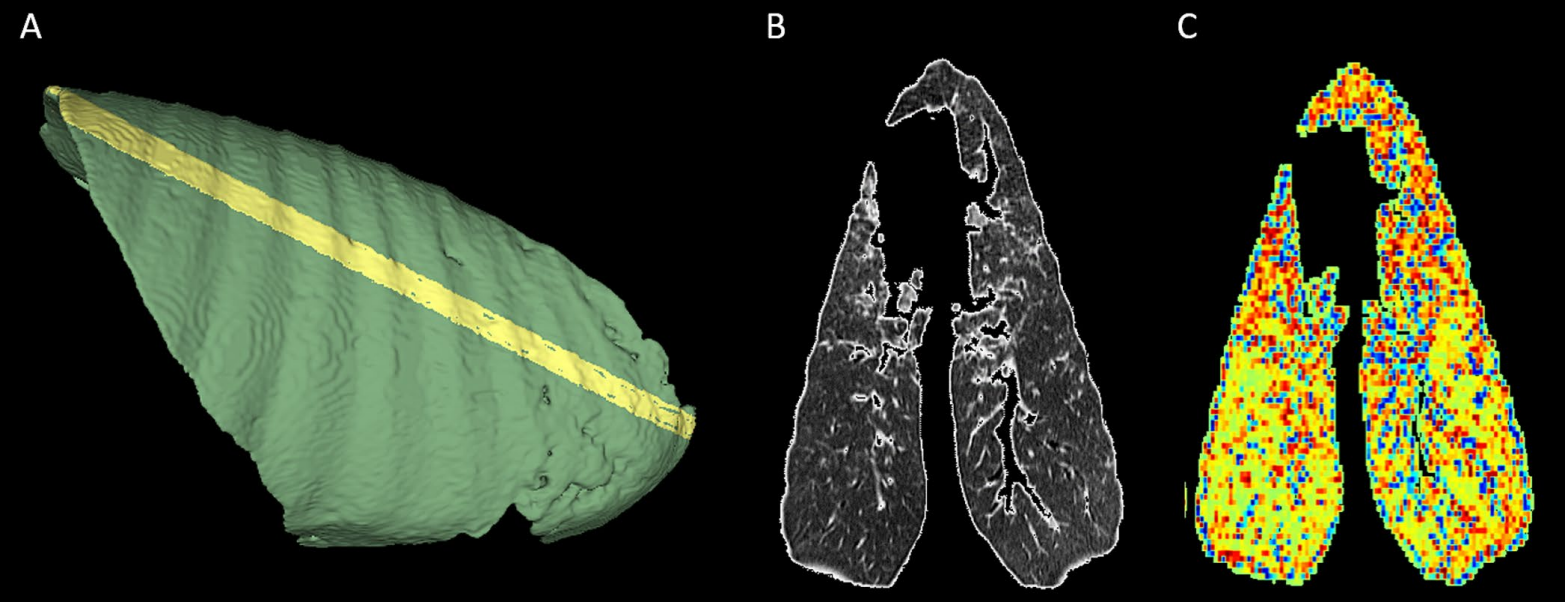
Feature map of glcm_contrast, demonstrating caudodorsal to cranioventral gradient. An oblique segmentation was created and used to mask and crop a randomly chosen lung volume from the cohort to facilitate radiomic feature map creation. **(A)** The oblique aspect of the segmentation is apparent on the pleural surface of the right lung. **(B)** The mid-slice CT image. **(C)** The related radiomic feature map for glcm contrast. An increasing caudodorsal to cranioventral intensity gradient is apparent for this feature. In **(B,C)**, the right lung is positioned on the right of the image, and the top of the image represents the cranial aspect.

Visual appraisal of these gradients was made possible through the generation of the radiomic feature maps for variables with a major influence on the principal components. A feature map of GLCM contrast, the texture feature with the strongest positive association with PC1, is shown in Figure 6.

### 3.5 Growth-related change in radiomic features

In the six sheep studied at monthly intervals between 4 and 12 months of age, bodyweight increased from 37.7 ± 3.1 to 67.5 ± 4.8 Kg and measured lung volumes increased from 1,297 ± 394 to 1840 ± 195 cm^3^ (mean ± SD).

**Figure 7:**
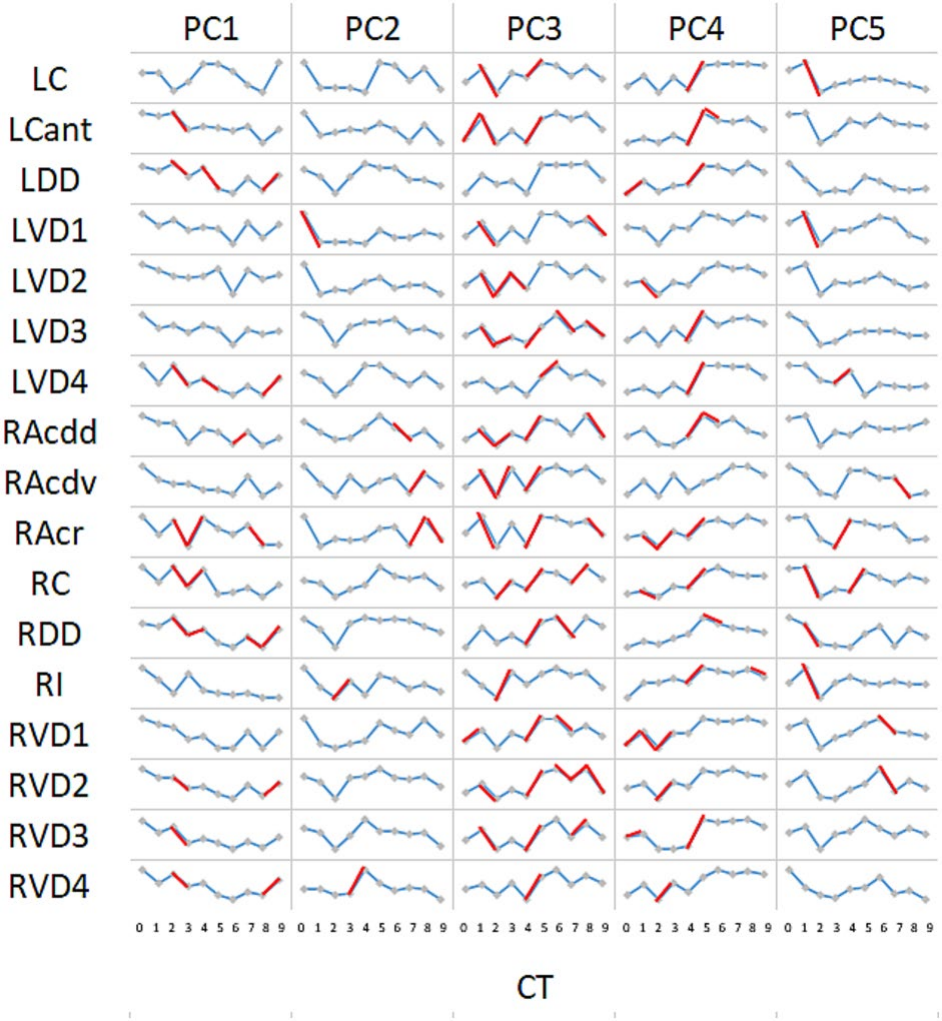
Time-dependent change in PCs by segment. For the majority of segments, a progressive decline in PC1 scores occurred throughout the time course. Notable exceptions to this pattern were apparent for LC and RAcr. For the remaining principal components, discerned patterns of dynamic change appeared largely consistent throughout the lung. Marker interconnections in red indicate where change between paired samples was significant (*p* < 0.05; Wilcoxon signed-rank test for paired data with the alternative hypothesis that the population median differs from the hypothesised median = 0).

**Figure 8:**
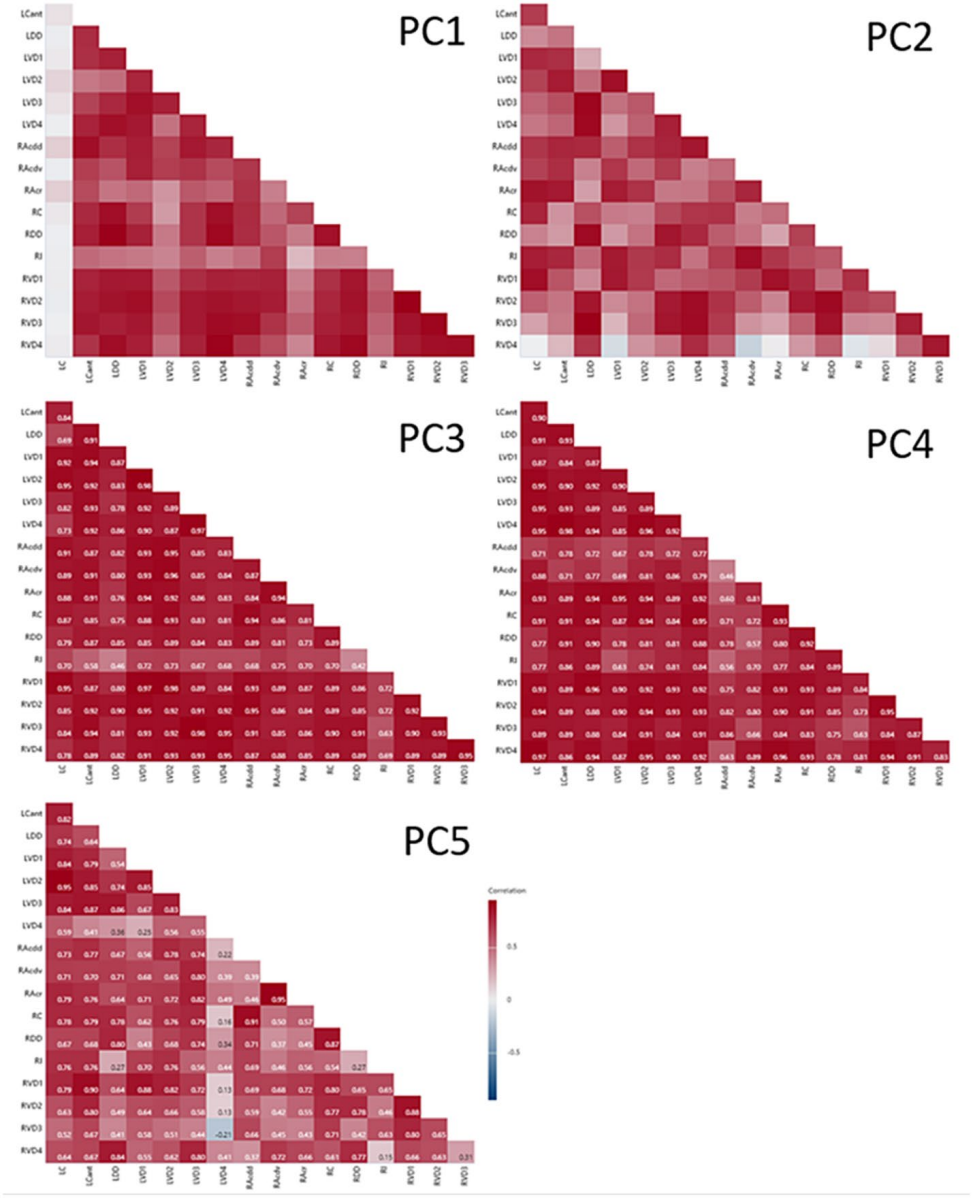
Correlograms of segmental PC values paired by CT. With the exception of LC for PC1, there was generally good agreement between segments for PC scores paired within CTs.

The time-dependent dynamics of segment PC scores are shown in Figure 7. Analysing the significance of change between consecutive CTs indicates that at times (in red) there was synchrony within the cohort of sheep; i.e., all six sheep demonstrated the same direction of change for the majority of segments (e.g., PC3 between CT1 and CT2, and between CT4 and CT5). The association between paired PC scores from different lung segments is illustrated in the correlograms in Figure 8. With the exception of LC for PC1 scores, there was generally a high degree of concordance between scores from different segments.

## 4 Discussion

In addressing our hypothesis, we used PCA to summarise the variation across the dataset and examined whether PCs would vary according to lung segment, reflecting a spatial ‘encoding’ of lung radiomic features. We demonstrated a region-specific increasing emphasis of PC1 from caudodorsal to cranioventral, with a positive bias towards the left lung, and RI notable as a positive extreme. A feature map prepared for GLCM contrast, the radiomic texture feature with the strongest positive association with PC1, confirmed this gradient. A marked decreasing dorsal to ventral gradient was noted for PC2 with particularly negative values for RC and LC. Regarding the observations that fuelled the central hypothesis, PC5 was notable for closely reflecting classic notions of respiratory pathology distribution associated with acute suppurative and acute fibrinous bronchopneumonia, as well as chronic bronchopneumonia (26). Such lung pathologies are generally multifactorial and often encompass infection with *Mannheimia haemolytica*, *Pasteurella multocida*, *Histophilus somni*, and *Mycoplasma* spp. (26). Whilst analysis of individual features would have demonstrated similar associations, the functional or form-based underpinnings of either PCs or individual radiomic feature measurements are unknown, and investigation of such is beyond the scope of the present study. Therefore, for the purpose of addressing the hypothesis, we opted for the simplicity that PCA affords.

The bronchi serving RI and RC arise in common from the right main bronchus, with RI located in the mediastinal recess between the heart and diaphragm. It was occasionally noted that the margins of RI were poorly defined following the lung segmentation. Whilst this effect was partly a consequence of the close association of this segment with the caudal cardiac and cranial diaphragmatic cupola margins, it was also compounded by the segment’s tendency towards increased attenuation relative to the other segments (RI -492 (−928, −15); remaining lung −646 (−794, −149); median (range) Hounsfield units). Special emphasis was placed on evaluating RI segment boundaries, and where necessary, manually adjusting them to compensate for this effect. However, it should be noted that conducting the PCA without including the RI segment led to the same general conclusions regarding the spatial segmental disposition of the major PCs.

Whilst the present approach averaged lung segmental radiomic feature values, Yang et al. (19), by employing a 3D sliding window kernel to capture radiomic features for each voxel, were able to generate 3D feature maps that revealed subsegmental variation in radiomic features that were otherwise visually inapparent (19). Such an approach could potentially offer more focus on exploring the lung pathobiology underlying particular radiomic feature extremes in preclinical models, where tissue sampling could be specifically directed by radiomic feature analysis of CTs acquired immediately prior to euthanasia.

Since our results indicate that radiomic features vary across the normal ovine lung, the question arises as to which aspects of lung function or anatomy might be associated with such PC distribution and, conceptually, with disease susceptibility. Certainly, the proportion of air relative to tissue is reduced in dependent lung regions, a feature discernible in CT as increased attenuation (27–29). Furthermore, studies of ventilation and perfusion in the prone sheep lung indicate that dependent (ventral) regions are relatively poorly ventilated, whereas perfusion is preferentially distributed to dorsal regions (30). Regional lung compliance maps obtained from anaesthetised supine pigs also highlight increased compliance in dependent lung regions (31), and varying elastic behaviour of lung tissue according to gravitational influence (32).

Changes in the healthy lung microbiome are an inherent feature of diseases such as suppurative bronchopneumonia or fibrinous pneumonia in small ruminants. We have previously demonstrated the variability that can exist in the healthy sheep lung microbiome with craniocaudal clustering evident (33), likely reflecting region-specific biochemical and physical pressures shaping ecosystem ecologies. The availability of multimodal spatial omics technologies to analyse metabolites, histology, and gene expression across tissue regions (34) will help elucidate the underlying mechanisms shaping such relationships in lung health, and provide the foundation to understand disease susceptibility. As spatial radiomics offers the potential to characterise *in vivo* subregional lung features relevant to specific aspects of lung biology and function, it will be a significant partner in this regard.

We found that, with the notable exceptions of LC and RAcr, the majority of segments demonstrated a progressive decline in PC1 scores between 4 and 12 months of age, a period of time when measured lung volumes increased appreciably. In lambs, postnatal lung volume changes are rapid during the first 2 months of life, accommodating the growth of nearly half the number of alveoli found in adult sheep, as well as an increase in alveolar size. Thereafter, the rate of increase starts to plateau, with volume increases beyond 6 months of age being much more modest (35). In line with the overall growth in lung volume, the pulmonary capillary network continues to expand through adulthood (36). Changes in the mechanical properties of the lung are evidenced by studies in goats demonstrating growth-related increases in dynamic lung compliance and viscous work of breathing and a decrease in total pulmonary resistance between 20 and 550 days of age (37). Whilst it is not known whether the major PC is specifically influenced by any of these changes, it is, however, tempting to speculate that the progressive change in PC values does indeed reflect on some aspects of growth-related changes in lung structure and/or function.

For the remaining PCs, discerned patterns of dynamic change appeared largely consistent throughout the lung. In some instances (e.g., PC3 between CT1 and CT2 and between CT4 and CT5), the monthly patterns of change were consistent (i.e., demonstrated by every sheep in the group). These sheep were group housed within the same airspace and demonstrated no clinical signs suggestive of respiratory disease throughout the time course of the experiment. However, our analysis indicates that every sheep experienced the same pattern of change in their lung CT images at the same point in time. The radiomic features with the strongest positive association to PC3 were GLRLM low grey-level run emphasis, a measure of the distribution of low grey-level values, and GLDM large-dependence low grey-level emphasis, which reflects the grey-level relationship between a central pixel or voxel and its neighbourhood (Table 1). Both reflect low-resolution lung structure heterogeneity, i.e., coarse texture (38, 39). These healthy co-habiting animals experiencing similar subtle changes in lung image characteristics are significant and warrant further analysis. In particular, probing for associations with more sensitive indices of systemic and respiratory states, as well as air quality in the housing environment, such as the presence of particulates and bioaerosols (airborne viruses, bacteria, and fungi), would appear logical.

In conclusion, we used PCA to reduce a dataset of lung radiomic features derived from healthy sheep lung segments to five uncorrelated PCs explaining 90% of the total variance. We established that the PCs are spatially encoded in the sheep lung, demonstrating an increasing emphasis of the first component from caudodorsal to cranioventral, and a marked decreasing dorsal-to-ventral gradient for PC2. Furthermore, we demonstrated a time-dependent progressive decline in PC1 scores between 4 and 11 months of age. Whilst patterns of change for PCs were generally highly consistent between segments, indicating an organ-level response, deviation from this was observed for the left cardiac segment, indicating a local influence on radiomic features. Finally, at certain times we observed the same patterns of change in texture features in all sheep group-housed together, suggesting a shared subclinical lung response to an unknown stimulus. Our findings provide a baseline for understanding the nature and temporal variation of radiomic features in healthy sheep lungs. Such data will be central to interpreting radiomic feature characteristics associated with disease and determining the extent of association between such changes and concomitant lung pathobiology.

## Data Availability

The raw data supporting the conclusions of this article will be made available by the authors, without undue reservation.

## Footnotes

^1^ www.slicer.org
